# The relationship between anxiety and dysfunctional breathing among the Russian population during the COVID-19 pandemic

**DOI:** 10.1192/j.eurpsy.2022.655

**Published:** 2022-09-01

**Authors:** J. Koniukhovskaia, E. Pervichko, O. Mitina, O. Stepanova, V. Petrenko, I. Shishkova, E. Dorokhov

**Affiliations:** 1 Pirogov Russian National Research Medical University, Clinical Psychology Department, Moscow, Russian Federation; 2 Lomonosov Moscow State University, Psychology, Moscow, Russian Federation; 3 Ryazan State Medical University named after I.P. Pavlov, Faculty Of Clinical Psychology, Ryazan, Russian Federation

**Keywords:** Anxiety, Covid-19 pandemic, dysfunctional breathing

## Abstract

**Introduction:**

Dysfunctional breathing is a pattern of respiratory movements that do not correspond to the physiological needs of the body and can lead to a series of respiratory, cardiovascular, digestive, sensory and neurological symptoms ( Vidotto et al., 2019). The causes of dysfunctional breathing are a combination of biological, psychological and social factors.

**Objectives:**

To examine the relationship between anxiety and occurrence of dysfunctional breathing in the Russian population under the conditions of the COVID-19 pandemic.

**Methods:**

We used a socio-demographic questionnaire, the Naimigen questionnaire (Van Dixhorn, Duivenvoordent, 1985), the State-Trait Anxiety Inventory (Spielberger et al., 1983). The study was conducted online from April 27 to December 28, 2020. It was attended by 1,362 people from all regions of Russia, including 1,153 women and 209 men aged 15 to 88 years (38.3 ±11.4)

**Results:**

It was revealed that with a low level of state anxiety (< 35 points), dysfunctional breathing was detected in 4.8% of respondents; while with a borderline level of anxiety (> 60 points) there were at 55.9%. A similar dependence was found for personal anxiety: at a low level (< 35 points), dysfunctional breathing was detected in only 4% of respondents;while at a borderline level of anxiety (> 60 points) at 62.8%.

**Conclusions:**

Dysfunctional breathing can occur among people with high and borderline levels of situational and personal anxiety during the COVID-19 pandemic. Results allows us to conclude that dysfunctional breathing and anxiety are not equivalent concepts, although they have a common phenomenological field. The study was supported of the Russian Science Foundation, project No. 21-18-00624.

**Disclosure:**

The study was supported of the Russian Science Foundation, project No. 21-18-00624.

